# The Prognostic Value of Neutrophil-to-Lymphocyte Ratio on Mortality in Patients Undergoing Transcatheter Aortic Valve Implantation: A Systematic Review and Meta-Analysis

**DOI:** 10.7759/cureus.77909

**Published:** 2025-01-24

**Authors:** Anurag Rawat, Priya Goyal, Syed Ali Ahsan, Krovvidi Syama Surya Srivyshnavi, Abdul Hannan Asghar, Abdallah A Riyalat, Calvin R Wei, Areeba Khan

**Affiliations:** 1 Interventional Cardiology, Himalayan Institute of Medical Sciences, Dehradun, IND; 2 Internal Medicine, Dayanand Medical College and Hospital, Ludhiana, IND; 3 Medicine, King Edward Medical University, Lahore, PAK; 4 School of Medicine, Akaki Tsereteli State University, Kutaisi, GEO; 5 Medicine, Quaid e Azam Medical College, Bahawalpur, PAK; 6 Pediatric Medicine, Sidra Medicine, Doha, QAT; 7 Research and Development, Shing Huei Group, Taipei, TWN; 8 Critical Care Medicine, United Medical and Dental College, Karachi, PAK

**Keywords:** meta-analysis, mortality, neutrophil-to-lymphocyte ratio, systemic inflammation, transcatheter aortic valve implantation

## Abstract

Transcatheter aortic valve implantation (TAVI) has emerged as a revolutionary treatment for severe aortic stenosis, but predicting post-procedure outcomes remains challenging. This systematic review and meta-analysis investigated the association between neutrophil-to-lymphocyte ratio (NLR) and mortality in TAVI patients. We searched major databases from January 2010 to November 2024, including studies examining pre-procedural NLR and mortality outcomes in TAVI patients. Eight retrospective studies comprising 9,948 participants were included. The follow-up duration ranged from three to 36 months. Meta-analysis using a random-effects model revealed that elevated NLR was significantly associated with increased mortality risk (RR: 1.37, 95% CI: 1.08-1.74). Substantial heterogeneity was observed (I-square: 77.3%). Meta-regression analysis identified follow-up duration as a significant predictor explaining 62.5% of the heterogeneity, while other variables including age, gender, diabetes, and hypertension showed minimal impact. Sensitivity analyses demonstrated the robustness of findings, with no individual study significantly influencing the overall effect size. The association between elevated NLR and increased mortality risk may be explained by neutrophils' role in inflammatory responses and tissue damage, coupled with reduced lymphocyte-mediated immune regulation. Despite limitations including NLR cut-off variations and heterogeneity in study designs, our findings suggest that NLR serves as a valuable prognostic marker for mortality risk in TAVI patients. Future research should focus on standardizing NLR thresholds and exploring subgroup-specific effects to enhance its clinical utility.

## Introduction and background

Transcatheter aortic valve implantation (TAVI) has revolutionized the management of severe aortic stenosis, offering a minimally invasive alternative to traditional surgical aortic valve replacement, particularly for patients at high surgical risk [1]. Despite advancements in TAVI techniques and technology, predicting outcomes and identifying patients at increased risk of adverse events post-procedure remains a significant clinical challenge [2]. In recent years, systemic inflammation has emerged as a crucial determinant of prognosis in cardiovascular diseases, including TAVI, because it plays a pivotal role in both acute and chronic disease processes. Inflammation influences vascular remodeling, endothelial function, and immune responses, all of which are critical in cardiovascular health [3]. 

The neutrophil-to-lymphocyte ratio (NLR), a marker of systemic inflammation, has gained attention for its prognostic value in various clinical settings like cancer (e.g., lung, colorectal, and gastric cancer), infectious diseases (e.g., sepsis, COVID-19), chronic inflammatory diseases (e.g., rheumatoid arthritis, inflammatory bowel disease), acute coronary syndrome (ACS), and chronic kidney disease (CKD). In these settings, elevated NLR is associated with poorer outcomes, highlighting its potential as a prognostic marker across multiple clinical scenarios [4]. NLR is calculated by dividing the number of neutrophils by the number of lymphocytes in a patient's blood, providing a simple yet insightful indicator of the balance between pro-inflammatory and anti-inflammatory responses [5]. Elevated preoperative NLR values have been associated with increased morbidity and mortality in cardiac surgery patients [6]. However, the predictive power of NLR on outcomes specifically in patients undergoing TAVI, particularly focusing on mortality, is less well-characterized and warrants further investigation [7-8]. Systemic inflammation plays a critical role in the pathophysiology of adverse outcomes following TAVI. Factors such as periprocedural myocardial injury, vascular trauma, and the host response to bioprosthetic valves may trigger an exaggerated inflammatory state, contributing to complications such as heart failure, arrhythmias, and valve dysfunction [9]. NLR, as a composite marker of inflammation and immune status, could provide insights into the underlying mechanisms linking inflammation to poor clinical outcomes [10-11]. 

Predicting adverse events such as valve dysfunction, mortality, and heart failure post-TAVI remains challenging due to the heterogeneity of patient profiles, including advanced age and comorbidities [8]. Systemic inflammation is uniquely significant in TAVI compared to other cardiovascular procedures because it exacerbates procedural stress, affects tissue healing, and impacts bioprosthetic valve durability [9-11]. This inflammation often reflects pre-existing conditions or procedural factors, influencing outcomes. Research in this area is complicated by conflicts such as variations in NLR thresholds, inconsistent definitions of key outcomes, and differences in follow-up durations. These discrepancies hinder the development of standardized predictive models for post-TAVI events.

While individual studies have reported conflicting results regarding the prognostic value of NLR in TAVI patients, a systematic evaluation of the available evidence is essential to establish its role as a reliable marker of mortality risk. A meta-analytic approach allows for the synthesis of data from diverse populations and methodological contexts, providing a more robust and generalizable assessment. 

This systematic review and meta-analysis aim to evaluate the effect of NLR on mortality in patients undergoing TAVI. Specifically, it seeks to determine whether elevated pre- or postprocedural NLR is associated with increased short- and long-term mortality risk, and to explore potential heterogeneity in this association across different patient subgroups and study designs. Understanding the prognostic significance of NLR may have important implications for risk stratification, early intervention, and improving outcomes in TAVI patients. 

## Review

Methodology 

Information Sources and Search Strategy 

A comprehensive literature search was conducted from January 1, 2010, to November 30, 2024, across multiple electronic databases, including PubMed (MEDLINE), Scopus, Web of Science, and EMBASE. The search strategy was designed to capture all relevant studies examining the relationship between NLR and mortality in patients after TAVI, combining Medical Subject Headings (MeSH) terms and keywords: ("Neutrophil to Lymphocyte Ratio" OR "NLR" OR "Systemic Inflammation") AND ("Transcatheter Aortic Valve Implantation" OR "TAVI" OR "Aortic Valve Replacement, Transcatheter") AND ("Mortality" OR "Survival" OR "Outcome"). Additionally, hand searching of relevant journals, screening of reference lists from included studies, and a grey literature search were performed to minimize the risk of omitting pertinent studies. The search was performed by two authors independently. Any disagreement between two authors was resolved through discussion. 

Eligibility Criteria and Study Selection 

Studies were deemed eligible for inclusion if they met specific criteria: focusing on adults undergoing TAVI, examining pre-procedural NLR, and reporting mortality as an outcome, with eligible study designs including observational studies (cohort, case-control) irrespective of the duration of follow-up. Exclusion criteria consisted of non-English publications, inaccessible full-texts, studies not reporting mortality, and non-original research such as reviews, editorials, case reports, or conference abstracts without corresponding full-text publications. The screening process involved two independent reviewers assessing titles and abstracts, resolving discrepancies through consensus or consultation with a third reviewer, followed by full-text evaluation of potentially eligible studies. 

Data Extraction and Quality Assessment 

Data extraction was meticulously performed using a pre-designed spreadsheet, capturing essential study characteristics (e.g., author, year, sample size, region, and NLR measurement) and outcomes. The methodological quality and risk of bias of included studies were assessed utilizing the Newcastle-Ottawa Scale (NOS) for observational studies facilitating the evaluation of the evidence's reliability and robustness. 

Data Analysis 

All statistical analyses were conducted using RStudio (Posit PBC, Boston, MA, USA) using the R *meta *package (R Foundation for Statistical Computing, Vienna, Austria), with the primary objective of synthesizing the association between pre-procedural NLR and mortality in TAVI patients. The meta-analysis employed a random-effects model to account for between-study heterogeneity, quantifying the pooled effect size via odds ratios (OR) with corresponding 95% confidence intervals (CI). We used the Knapp-Hartung adjustment to calculate CI as this methodology is usually applied for smaller sample sizes, thus, providing more conservative confidence intervals, reinforcing result reliability. Heterogeneity was assessed using the I-square statistic, with sensitivity tests planned to explore potential sources of heterogeneity and the robustness of the findings, respectively. We used leave-out sensitivity analysis to assess how the exclusion of any study impacts the overall effect of NLR on mortality.

Results 

**Figure 1:**
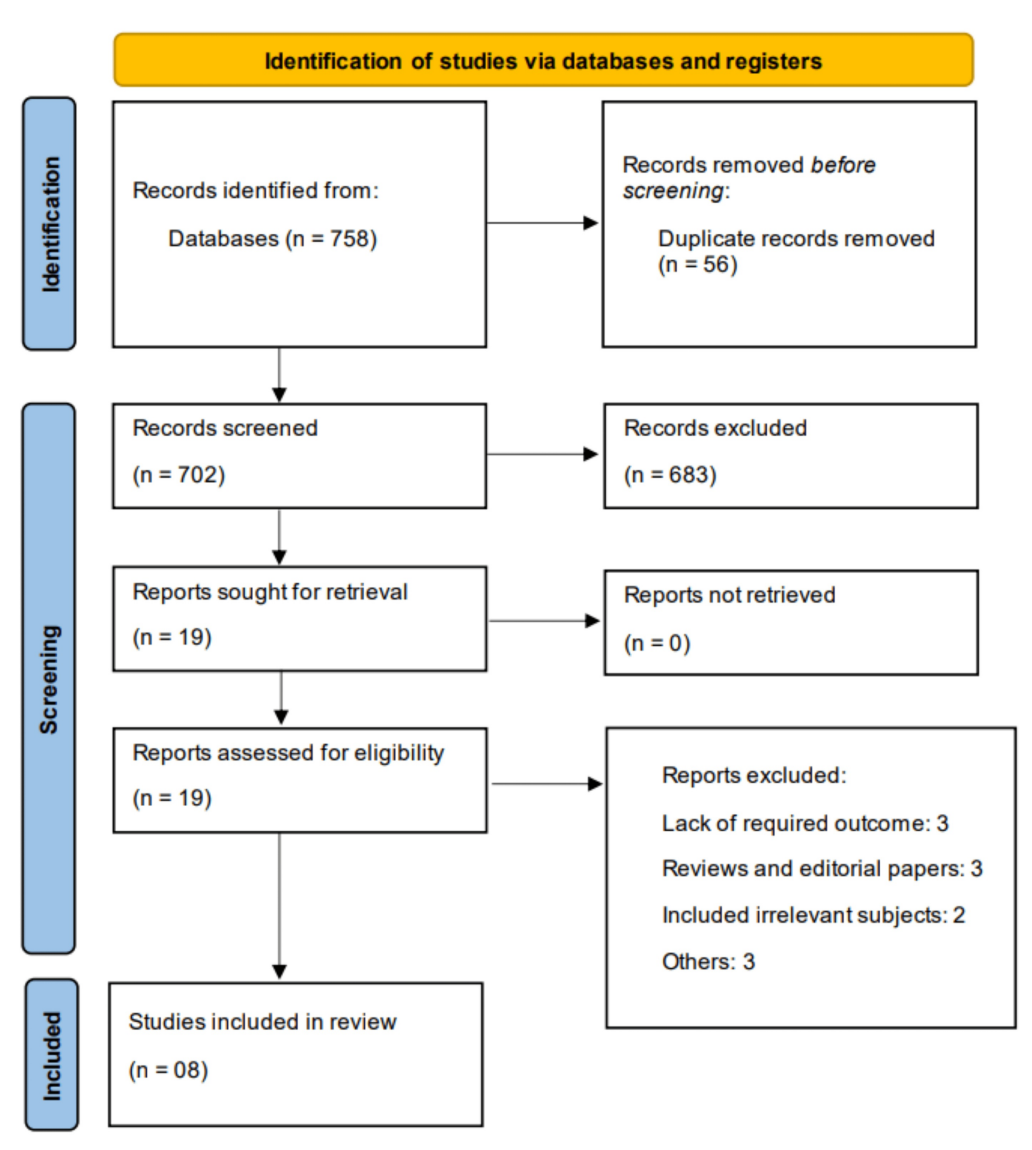
Preferred Reporting Items for Systematic Reviews and Meta-Analyses (PRISMA) flow showing study selection process

From electronic databases, we identified 758 records. After removing duplicates, 702 studies were screened using their titles and abstracts. Full text of 19 studies was obtained and detailed assessment of their eligibility criteria was done. Finally, eight studies were included in this meta-analysis. A detailed description of how the studies were selected is presented in Figure 1. 

Study Characteristics 

The characteristics of individual studies are shown in Table 1. Eight studies including 9948 participants reported the relationship of NLR and mortality. Follow-up of individual studies ranged from three months to 36 months. All included studies were retrospective. Four studies were conducted in the United States, two in Australia, and one each in Israel and China. 

**Table 1:** Included studies characteristics NR: Not reported

Author	Region	Study Design	Sample Size	Type of Measure	Follow-up duration	age	Male	Diabetes	Hypertension
Abushouk et al., 2022 [12]	United States	Retrospective	908	Adjusted	30 Months	NR	NR	NR	NR
Condado et al., 2013 [13]	United States	Retrospective	520	Adjusted	12 Months	83	263	219	501
Gao et al., 2024 [14]	China	Retrospective	124	Adjusted	3 Months	79.07	62	36	81
Habib et al., 2018 [15]	United States	Retrospective	335	Adjusted	36 Months	70.61	136	84	186
Merdler et al., 2022 [16]	Israel	Retrospective	1152	Adjusted	36 Months	82.9	509	419	987
Nair et al., 2022 [17]	Australia	Retrospective	304	Adjusted	17 Months	NR	NR	NR	NR
Nair et al., 2024 [7]	Australia	Retrospective	367	Adjusted	12 Months	84	189	115	289
Shahim et al., 2022 [18]	United States	Retrospective	5881	Adjusted	24 Months	83	3272	2118	5452

Effect of NLR on Risk of Mortality in Patients With TAVI 

**Figure 2:**
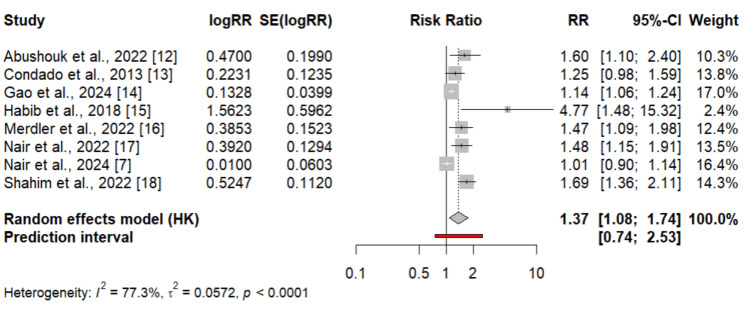
Comparing the effect of neutrophil-to-lymphocyte ratio (NLR) on mortality RR: Risk ratio; CI: Confidence interval; HK: Knapp-Hartung adjustment Sources: References [7,18]

**Figure 3:**
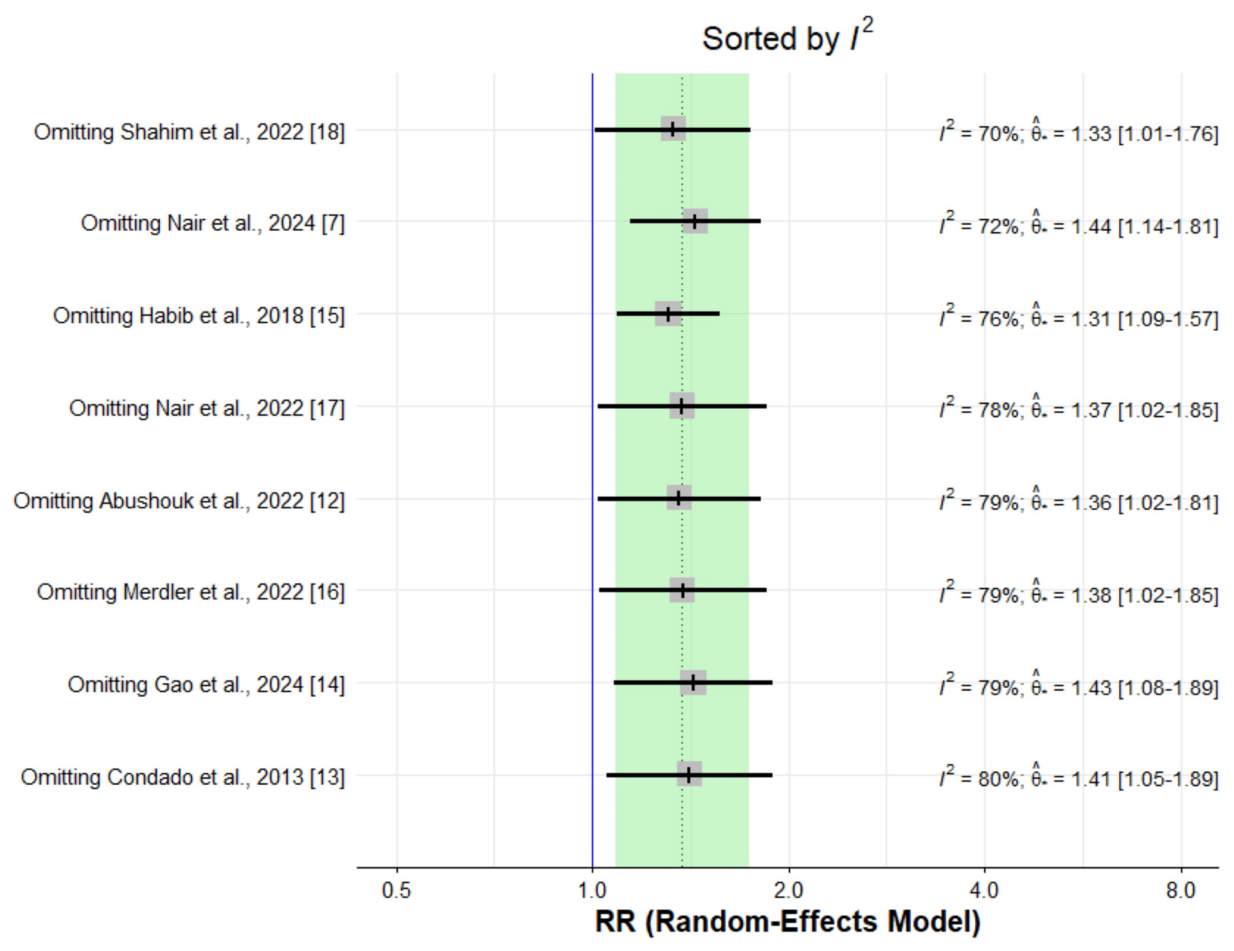
Sensitivity Analysis: Removing One Study at a Time RR: Risk ratio

Figure 2 presents the pooled analysis assessing the impact of high NLR on all-cause mortality in patients undergoing TAVI. Pooled analysis of eight studies showed that high NLR is associated with increased risk of mortality (RR: 1.37, 95% CI: 1.08 to 1.74). High heterogeneity was reported among the study results (I-square: 77.3%). To identify the study causing high heterogeneity among the study results, we performed leave-one-out analysis leaving one study at a time. The results of the sensitivity analysis are shown in Figure 3. This forest plot shows that omitting any individual study from the meta-analysis has minimal impact on the overall effect size (RR), with I² values remaining consistently high (70-80%) across all leave-one-out analyses. 

To identify whether any study is an outlier, i.e. defined as any study that shows a substantial influence on the model results when removed, where "substantial" typically means causing a shift in the combined effect estimate outside the original confidence interval, we used "find.outliers" function within "dmetar" in R. We did not identify any outlier study. This finding is supported by our leave-one-out analysis Figure 3, which demonstrates that removing individual studies did not substantially alter the pooled effect estimate, with the estimates remaining stable across all iterations. 

Meta-Regression 

Table 2 presents the results of the meta-regression analysis examining the effect of various predictors on the relationship between NLR and mortality. The year of publication does not significantly impact this relationship, as indicated by a non-significant p-value and an R-square value of 0%, suggesting it does not account for any heterogeneity in the data. Follow-up duration, however, is a statistically significant predictor, with a positive estimate, and it explains a substantial portion of the heterogeneity (62.50%). Age, while showing a negative estimate, is not significantly associated with mortality outcomes, and it accounts for 4% of the heterogeneity. Gender (male), diabetes, and hypertension all show negligible effects on the NLR-mortality relationship, as evidenced by their non-significant p-values and R-square values of 0%, indicating that they do not explain any meaningful variation in the data. Overall, the meta-regression suggests that follow-up duration may be an important factor in the relationship between NLR and mortality, while other variables like age, gender, diabetes, and hypertension appear to have little impact. 

**Table 2:** Results of Meta-Regression Analysis: Predictors of Study Outcomes R-Square: amount of heterogeneity accounted for

Variable	Estimate	P-value	R-Square
Year of publication	-0.0143	0.6658	0%
Follow-up duration	0.0134	0.0365	62.50%
Age	-0.06	0.2269	4.00%
Gender (Male)	0.0001	0.5941	0%
Diabetes	0.0001	0.5884	0%
Hypertension	0.0001	0.5937	0%

Effect of Follow-Up Duration on Relationship Between NLR and Mortality

**Figure 4:**
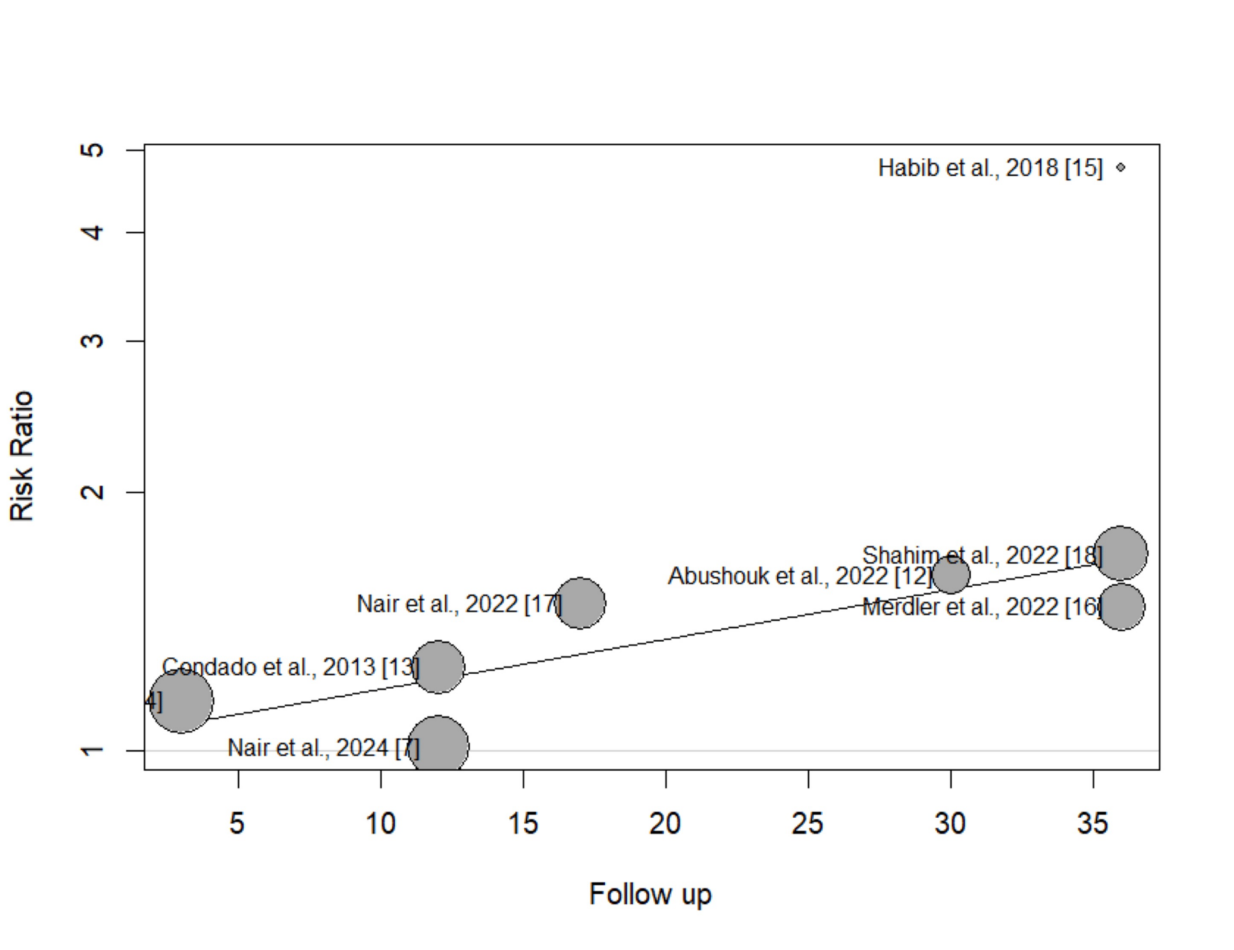
Bubble plot assessing the effect of follow-up on study outcome

This bubble plot in Figure 4 displays the relationship between follow-up duration (x-axis) and risk ratio (y-axis) across different studies, where the size of each bubble represents the study's weight in the meta-analysis. There appears to be a slight positive trend line, suggesting that the risk ratio tends to increase with longer follow-up periods. Notably, Habib et al. (2018) appears as an outlier with a much higher risk ratio (~4.5) compared to other studies as the study follow-up is larger, while most studies cluster between risk ratios of 1 to 2. 

Discussion 

Our meta-analysis, which included eight cohort studies, demonstrates that the NLR is a significant predictor of all-cause mortality in patients undergoing TAVI. Patients with elevated NLR had a notably higher risk of mortality, with an RR of 1.37 (95% CI: 1.08 to 1.74). Similarly, a meta-analysis by Wang et al., which encompassed 10 cohort studies, found that NLR is a reliable predictor of both all-cause mortality and major adverse cardiovascular events (MACE) in individuals undergoing angiography or cardiac revascularization. In their analysis, patients in the highest NLR category faced a significantly higher risk of mortality (RR: 2.33, 95% CI: 1.88-2.88) and cardiovascular events (RR: 1.89, 95% CI: 1.42-2.52) compared to those in the lowest NLR category. These findings further support the role of NLR as an important biomarker for predicting adverse outcomes in cardiovascular patients. 

Several factors could explain why an increased NLR is associated with higher all-cause mortality and cardiovascular events. Neutrophils play a critical role in the inflammatory response to acute myocardial injury through various biochemical mechanisms, which can contribute to further tissue damage. These mechanisms include the release of reactive oxygen species, myeloperoxidase, and proteolytic enzymes, which facilitate plaque disruption [19]. In contrast, lymphocytes are involved in regulating the immune system's response [20-22]. However, inflammation has been shown to promote lymphocyte apoptosis, reducing their effectiveness in controlling the immune response [23]. Thus, the combination of elevated neutrophil levels and reduced lymphocyte counts into a single composite marker of inflammation, such as NLR, can provide valuable prognostic information. This imbalance may contribute to an increased risk of adverse outcomes, including all-cause mortality and cardiovascular events, highlighting the importance of NLR as a marker of inflammatory status in cardiovascular disease [19]. 

NLR is an unspecific indicator of systemic inflammation, reflecting the ratio between two distinct types of leukocytes: neutrophils, which are involved in the innate, non-specific immune response, and lymphocytes, which play a role in the adaptive, regulatory immune pathway. A key challenge in utilizing NLR as a prognostic tool in disease processes is identifying an optimal cut-off value that differentiates disease outcomes. This cut-off value is likely to vary depending on factors such as the stage of the illness, the laboratory methods used, concurrent infections, and the demographic characteristics of the patient population [24]. 

Several government-approved clinical trials are currently exploring the role of NLR in various cardiovascular diseases and publishing their findings in reputable scientific journals. One such example is the Prospective Multicenter Observational Study of Patients with Heart Failure with Preserved Ejection Fraction (UMIN000021831), which included 1,026 participants. In this study, elevated levels of NLR (>4.5) at admission were independently linked to an increased risk of cardiac death [25]. An earlier study (NCT01663194) aimed to demonstrate the relationship between long-term outcomes for ST-elevation myocardial infarction (STEMI) patients undergoing percutaneous coronary intervention (PCI) and pre-procedural NLR in the hospital [26]. 

The current meta-analysis revealed substantial heterogeneity across the study results, highlighting significant variation in the relationship between NLR and mortality. Despite all the studies showing a positive association between NLR and mortality, there were differences in the magnitude of this effect, suggesting that other factors might be contributing to the observed variability. To explore potential sources of this heterogeneity, we conducted a meta-regression analysis, focusing on various study characteristics that could influence the NLR-mortality relationship. Our findings suggest that the follow-up duration is a key factor explaining the high level of heterogeneity. Studies with longer follow-up periods were associated with a higher reported mortality rate, which may reflect the cumulative effect of NLR on mortality over time. In fact, follow-up duration accounted for 62.5% of the heterogeneity observed in the studies. This suggests that the length of time over which mortality is assessed plays a crucial role in moderating the observed effect of NLR on mortality outcomes. Longer follow-up periods may capture more mortality events and reflect cumulative inflammatory burdens, with chronic inflammation potentially altering the relationship between NLR and outcomes [16]. Additionally, changes in baseline inflammatory markers over time could amplify NLR’s predictive strength. 

This meta-analysis acknowledges several limitations. One of the primary concerns is the variation in the levels of NLR across the studies included, which may contribute to inconsistencies in the overall findings. Furthermore, substantial heterogeneity was observed in the pooled analysis of all-cause mortality. The majority of the studies were retrospective observation, which is associated with potential selection bias. While we were able to identify the main source of this heterogeneity through sensitivity analyses and meta-regression, the inability to conduct subgroup analyses due to insufficient data remains a limitation. This lack of subgroup data prevents a deeper exploration of the factors that could further explain the observed variability in the NLR-mortality relationship. Lastly, the majority of these studies were conducted in the United States and other developed countries and cannot be generalized to populations in low- and middle-income countries. Despite these limitations, the study provides valuable insights into the association between NLR and mortality, though future research with more uniform data and subgroup analyses would help clarify the underlying mechanisms and refine the conclusions. 

## Conclusions

This meta-analysis of eight studies encompassing 9,948 TAVI patients demonstrates that elevated NLR is significantly associated with increased mortality risk (RR: 1.37, 95% CI: 1.08-1.74). Follow-up duration emerged as a crucial moderator, explaining 62.5% of the observed heterogeneity. The relationship between elevated NLR and mortality is likely mediated through inflammatory mechanisms, with neutrophils promoting tissue damage while reduced lymphocytes impair immune regulation. Despite study limitations, NLR shows promise as a practical prognostic marker for TAVI patients. Future research should focus on standardizing NLR thresholds and investigating subgroup-specific effects to enhance its clinical application.
